# Nomograms based on SIRI for predicting postoperative survival outcomes in patients with non-metastatic clear cell renal cell carcinoma

**DOI:** 10.1186/s12893-025-03349-y

**Published:** 2025-12-24

**Authors:** Baochao Zhang, Wenming Ren, Tao Yang, Junhai Fan, Jie Han, Hui Yang

**Affiliations:** 1 Department of Urology, Nanjing First Hospital, Nanjing Medical University, Nanjing, Jiangsu 2310000 China; 2 Department of Urology, Taihe County People’s Hospital, the Taihe Hospital of Wannan Medical College, Taihe, Anhui 236600 China; 3 Department of Urology, the First Affiliated Hospital of Wannan Medical College, Wuhu, Anhui 241000 China

**Keywords:** SIRI, Prognostic indicator, Survival, Nomogram, CcRCC

## Abstract

**Objective:**

This study aimed to investigate the prognostic significance of the preoperative Systemic Inflammation Response Index (SIRI) and to develop predictive models for overall survival (OS), cancer specific survival (CSS), and metastasis free survival (MFS) in patients with non-metastatic clear cell renal cell carcinoma (ccRCC) after nephrectomy.

**Methods:**

We conducted a retrospective analysis of clinicopathological and prognostic data from 231 non-metastatic ccRCC patients. The optimal cutoff value for SIRI was determined using receiver operating characteristic (ROC) curve analysis. Prognostic factors were identified through least absolute shrinkage and selection operator (LASSO) regression and multivariable Cox proportional hazards models. Nomograms for predicting OS, CSS, and MFS were constructed based on selected predictors. The performance of the nomograms was evaluated using time-dependent ROC curves, time-dependent concordance index (C- index), calibration plots, and decision curve analysis (DCA). The predictive efficacy of our nomograms was compared with that of established models.

**Results:**

Among 231 patients, 21 (9.1%) died including 17 (7.4%) ccRCC-specific deaths; 32 (13.9%) developed postoperative metastases. An elevated SIRI (> 1.405) was independently associated with worse survival outcomes. Multivariable Cox analysis confirmed SIRI as an independent predictor of OS, CSS, and MFS. Nomograms integrating SIRI with other clinicopathological variables were successfully developed. Time dependent ROC curves and C - index demonstrated superior predictive performance of the nomogram compared to conventional clinicopathological characteristics. Calibration plots showed strong agreement between predicted and observed outcomes, and DCA confirmed high clinical utility. Our nomograms outperformed the established Stage, Size, Grade, and Necrosis (SSIGN) score and University of California Los Angeles Integrated Staging System (UISS) models in predictive accuracy.

**Conclusions:**

Elevated pretreatment SIRI independently predicts reduced OS, CSS, and MFS in non-metastatic ccRCC patients. The developed nomograms, which incorporate SIRI and key with clinicopathological characteristics, demonstrate excellent predictive performence, and serve as valuable tools for prognostic assessment in the management of patients with non-metastatic ccRCC.

**Supplementary Information:**

The online version contains supplementary material available at 10.1186/s12893-025-03349-y.

## Introduction

Renal cell carcinoma (RCC) represents the third most common and lethal genitourinary malignancy worldwide, with its global incidence increasing at an annual rate of approximately 2% [1]. Surgical resection remains the standard treatment for localized disease [2]. Clear cell RCC (ccRCC), which accounts for nearly 75% of all RCC subtypes, is associated with a considerable risk of recurrence. Approximately 30% of patients initially diagnosed with non-metastatic ccRCC eventually develop metastasis or local recurrence after curative surgery significantly impairing long-term survival [3]. This unpredictable clinical course highlights the urgent need for reliable prognostic tools to improve risk stratification.

Accumulating evidence underscores the critical role of systemic inflammation in tumor initiation, progression, and metastasis [4]. Levels of systemic inflammation, which can be conveniently assessed using peripheral blood biomarkers, such as monocytes, neutrophils, lymphocytes and platelets, have been correlated with survival outcomes in various malignancies, including RCC [4, 5]. Among these biomarkers, the systemic inflammation response index (SIRI), calculated as the counts of peripheral inflammation biomarkers, has emerged as a promising prognostic indicator across multiple malignancies [6–8]. In 2016, Qi et al. [9] first defined the SIRI as (neutrophil count × monocyte count)/lymphocyte count in 177 patients with advanced pancreatic cancer and palliative chemotherapy. They demonstrated that SIRI consistently predicted both time to progression (TTP) and overall survival (OS), whereas other established inflammatory indices such as the lymphocyte-to-monocyte ratio (LMR), neutrophil-to-lymphocyte ratio (NLR), and platelet-to-lymphocyte ratio (PLR) showed inconsistent prognostic performance across three independent cohorts. These findings suggest that SIRI may offer superior prognostic utility in oncology.

Although several studies have indicated a potential prognostic value for SIRI in RCC [10, 11], the evidence remains inconclusive, particularly regarding its association with metastasis-free survival (MFS). To address this gap, the present study aimed to three primary objectives: first, evaluating the independent prognostic significance of SIRI in non-metastatic ccRCC; second, developing and internally validate a SIRI-integrated nomogram for postoperative risk stratification; and third, assessing the clinical utility of this model to support individualized patient management.

## Materials and methods

### Patient selection and data collection

We conducted a retrospective cohort study of patients with ccRCC who underwent diagnostic confirmation and either radical or partial nephrectomy at our institution between October 2011 and November 2017. Inclusion criteria were patients diagnosed with ccRCC and underwent either radical or partial nephrectomy. Exclusion criteria were: (1) a history of other malignancies; (2) accidental mortality following nephrectomy; (3) radiographically confirmed preoperative metastasis; (4) incomplete clinicopathological data; and (5) loss to follow-up.

**Fig. 1:**
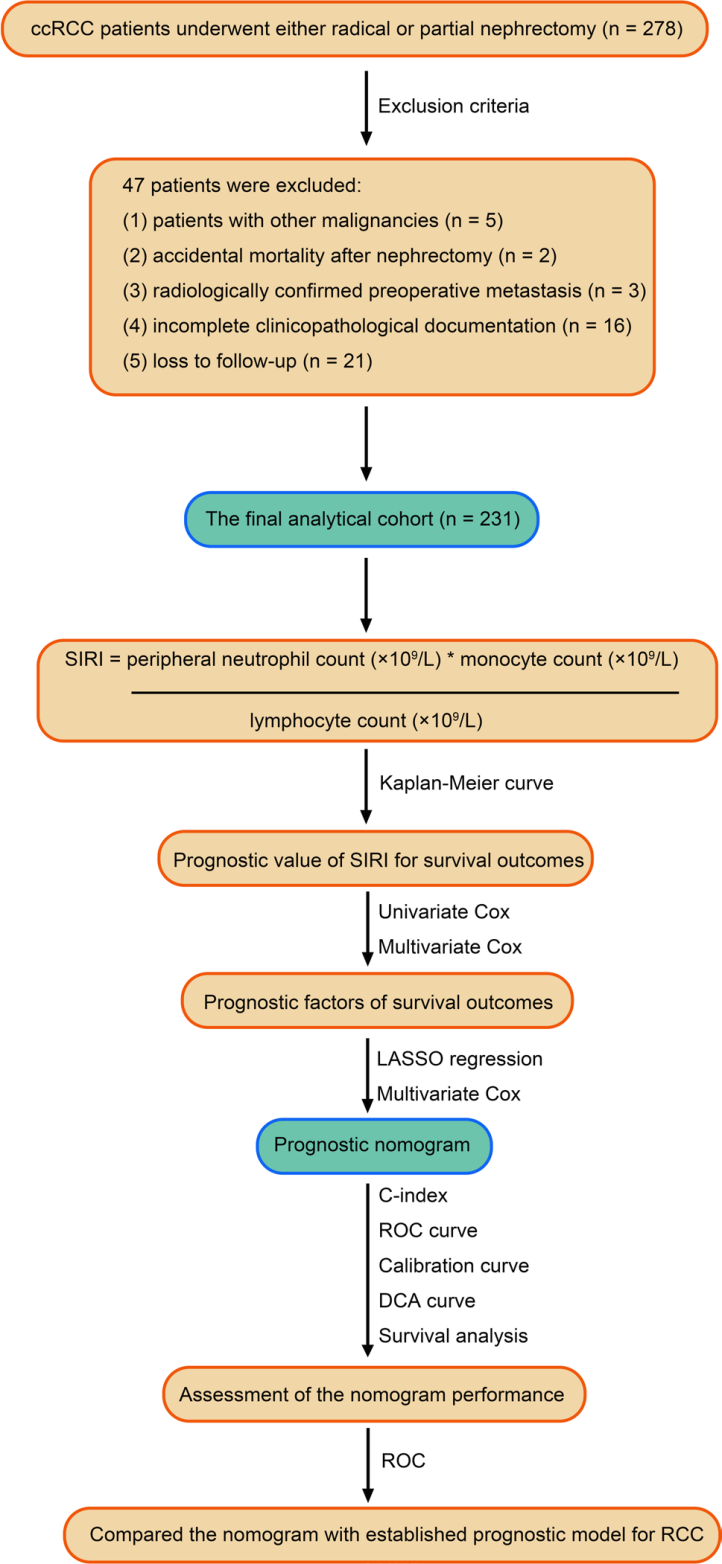
Flowchart of this design

After screening, 231 patients were included in the final cohort (Fig. 1). All surgeries were performed laparoscopically via transperitoneal or retroperitoneal approaches, and no patient received adjuvant targeted therapy during the follow-up period. Preoperative evaluation included standardized comprehensive laboratory profiling and contrast-enhanced renal computed tomography (CT) or magnetic resonance imaging (MRI) of the kidney for primary staging. When metastasis was suspected, additional imaging, such as thoracic CT, cerebral CT, bone scintigraphy, or positron emission tomography–CT (PET-CT), was conducted to rule out distant metastases before surgery.

Demographic and hematological parameters were retrieved from institutional electronic health records. The SIRI was calculated using the formula: [peripheral neutrophil count (×10⁹/L) × monocyte count (×10⁹/L)]/lymphocyte count (×10⁹/L). Histopathological evaluation was performed in accordance with contemporary standards: tumor staging followed the 8th edition American Joint Committee on Cancer (AJCC) TNM classification, and Fuhrman grade. All specimens were independently reviewed by three genitourinary pathologists who blinded to clinical outcomes.

Postoperative surveillance included annual clinical evaluations comprising physical examination, serum biomarker testing, and abdominal ultrasonography supplemented with CT or MRI. Survival status was recorded until December 31, 2022. The primary endpoints were: (1) OS: defined as the time from nephrectomy to death from any cause; (2) Cancer-specific survival (CSS): defined as the time from nephrectomy to death due to ccRCC; (3) MFS: defined as the time from nephrectomy to radiological or histopathological confirmation of distant metastases.

### Statistical analyses

All statistical analyses were performed using SPSS 26.0 (IBM SPSS Inc., Chicago, IL, USA) and R software v4.1.3 (R Foundation for Statistical Computing, Vienna, Austria). Continuous variables following a normal distribution are presented as mean ± standard deviation (SD) and were compared using independent samples t-tests. Non-normally distributed continuous variables are summarized as median (interquartile range, IQR) and were compared using Mann-Whitney U tests. Categorical variables are expressed as frequencies (%) and were analyzed with chi-square or Fisher’s exact tests, as appropriate. A two-sided *P* < 0.05 was considered statistically significant for all analyses.

### Determination and prognostic performance of the optimal SIRI cutoff value

The optimal cutoff value for SIRI was determined using time-dependent receiver operating characteristic (ROC) curve analysis based on Youden’s index, implemented with the R package “timeROC”. Discriminatory capacity was evaluated by the area under the ROC curve (AUC). Kaplan-Meier curves, supplemented with log-rank tests (conducted with R package “survival”), were used to compare OS, CSS, and MFS between low- and high-SIRI cohorts. Univariate and multivariate Cox proportional hazards analyses were performed using the R package “rms” to identify independent prognostic factors.

### Prognostic modeling development and validation

To construct the nomogram, we performed least absolute shrinkage and selection operator (LASSO) regression using the R package “glmnet”, applying the lambda.1se criterion to achieve an optimal balance between predictive accuracy and model simplicity. The 10-fold cross-validation method was applied to the iterative analysis. Model selection was conducted through 10-fold cross-validation, which identifies the most parsimonious model within one standard error of the minimum cross-validation error. Variables selected by the LASSO regression were incorporated into multivariate Cox proportional hazards models, and backward elimination was used to establish the final predictive nomogram.

The discriminative ability of the nomogram was assessed using the concordance index (C-index), computed with the R package survival, along with time-dependent receiver operating characteristic (ROC) curves. Calibration curves, generated using the R package “calibrationCurves”, were used to evaluate agreement between predicted probabilities and observed outcomes. Clinical utility was assessed via decision curve analysis (DCA) implemented with the R package “rmda”. Based on regression coefficient from the final model, each patient was assigned a prognostic score. Patients were then stratified into risk groups according to interquartile range of these scores. Survival differences among these groups were compared using Kaplan-Meier analysis with log-rank tests.

To further validate the nomogram, we compared its performance with two established prognostic models for RCC, the Stage, Size, Grade, and Necrosis (SSIGN) score and University of California Los Angeles Integrated Staging System (UISS) prognostic model, using time-dependent AUC metrics.

## Results

### Clinicopathological characteristics

Table 1 summarizes the clinicopathological characteristics of the 231 patients with non-metastatic ccRCC. By the end of the follow-up period, 21 patients (9.1%) had died from all causes, including 17 deaths (7.5%) attributed to ccRCC. Metastatic progression occurred in 32 patients (14.1%).

**Table 1:** Clinicopathological data of patients with non-metastatic CcRCC

Variables	Total (*n* = 231)	SIRI		*P* value
		Low (*n* = 164)	High (*n* = 67)	
Age (years, mean ± SD)	56.95 ± 11.55	57.01 ± 12.28	56.82 ± 9.61	***0.912***
Gender (n, %)				
Male	142(61.5%)	92 (39.8%)	50 (21.7%)	***0.013***
Female	89(38.5%)	72 (31.2%)	17 (7.3%)	
Hypertension (n, %)				
Yes	101(43.7%)	70 (30.3%)	31 (13.4%)	***0.725***
No	130(56.3%)	94 (40.7%)	36 (15.6%)	
Diabetes mellitus (n, %)				
Yes	25(10.8%)	17 (7.4%)	8 (3.4%)	***0.908***
No	206(89.2%)	147 (63.6%)	59 (25.5%)	
Coronary heart disease (n, %)				
Yes	17(7.4%)	11 (4.8%)	6 (2.6%)	***0.752***
No	214(92.6%)	153 (66.2%)	61 (26.4%)	
BMI (kg/m^2, median, IQR)^	21.22(23.33,25.61)	23.30 (21.22, 25.32)	23.53 (21.55, 26.17)	***0.240***
ASA score (n, %)				
1	13(5.6%)	9 (3.9%)	4 (1.7%)	***0.274***
2	177(76.6%)	126 (54.5%)	51 (22.1%)	
3	39(16.9%)	29 (12.6%)	10 (4.3%)	
4	1(0.4%)	0 (0.00)	1 (0.4%)	
5	1(0.4%)	0 (0.00)	1 (0.4%)	
Surgical approach (n, %)				
Partial nephrectomy	56(24.2%)	46 (19.9%)	10 (4.3%)	***0.052***
Radical nephrectomy	175 (75.8%)	118 (51.1%)	57 (24.7%)	
Tumor laterality (n, %)				
left	110(47.6%)	78 (33.8%)	32 (13.8%)	***1.000***
right	121(52.4%)	86 (37.2%)	35 (15.2%)	
Multiple tumors (n, %)				
Yes	5(2.2%)	3 (1.3%)	2 (0.9%)	***0.960***
No	226(97.8%)	161 (69.7%)	65 (28.1%)	
Tumor size (cm, ^median, IQR)^	4.00(3.00,5.50)	4.00 (3.00, 4.85)	4.80 (3.10, 7.00)	***0.001***
Pathologic T stage (n, %)				
T1-2	222(96.1%)	160 (69.3%)	62 (26.8%)	***0.157***
T3-4	9(3.9%)	4 (1.7%)	5 (2.2%)	
Fuhrman grade (n, %)				
Ⅰ-II	213(92.2%)	157 (68.0%)	56 (24.2%)	***0.004***
ⅡI-IV	18(7.8%)	7 (3.0%)	11 (4.8%)	
Tumor necrosis (n, %)				
Yes	9(3.9%)	6 (2.6%)	3 (1.3%)	***1.000***
No	222(96.1%)	158 (68.4%)	64 (27.7%)	
Tumor hemorrhage (n, %)				
Yes	28(12.1%)	21 (9.1%)	7 (3.0%)	***0.783***
No	203(87.9%)	143 (61.9%)	60 (26.0%)	
All-cause mortality (n, %)				
Yes	21 (9.1%)	7 (3.0%)	14 (6.1%)	***< 0.001***
No	210 (90.9%)	157 (68.0%)	53 (21.1%)	
Cancer-specific mortality (n, %)				
Yes	17 (7.5%)	6 (2.6%)	11 (4.9%)	***0.001***
No	210 (92.5%)	157 (68.0%)	53 (21.1%)	
Metastasis (n, %)				
Yes	32 (14.1%)	14 (6.1%)	18 (7.8%)	***< 0.001***
No	195 (85.9%)	149 (64.5%)	46 (19.9%)	

*SD* Standard deviation, *IQR *Interquartile range, *BMI *Body mass index, *ASA *American society of Anesthesiologists, *SIRI *Systemic inflammation response index

**Fig. 2:**
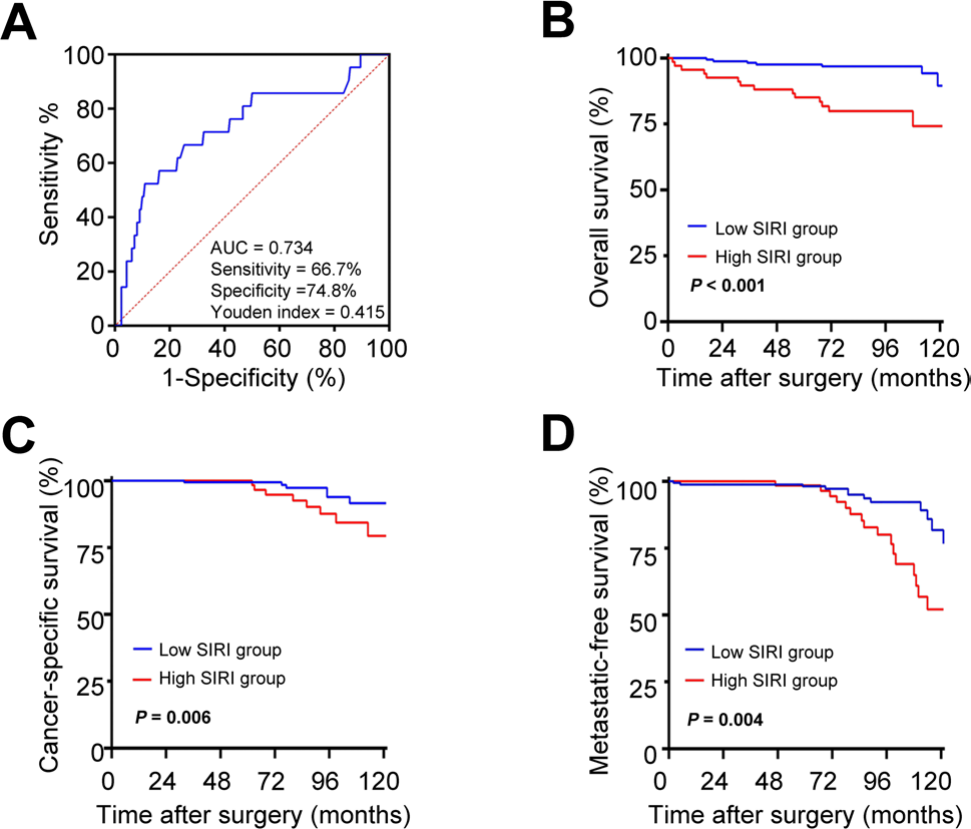
Prognostic evaluation of SIRI in patients with non-metastatic ccRCC (**A**) Time-dependent ROC analysis for determining the optimal SIRI cutoff value based on OS. (**B-D**) Kaplan-Meier survival curves comparing OS (**B**), CSS (**C**), and MFS (**D**) between high- and low-SIRI groups, stratified using the optimal cutoff derived from **A**. ccRCC, Clear cell renal cell carcinoma; SIRI, Systemic inflammation response index; OS, Overall survival; CSS, Cancer specific survival; MFS, Metastasis free survival; ROC, Receiver operating characteristic curve

The optimal cutoff value for the SIRI was determined using time-dependent ROC curve analysis (Fig. 2A). Using the optimal cutoff of 1.41, which yielded a sensitivity of 66.7% and a specificity of 74.8%, patients were categorized into low- and high-SIRI groups. Comparative analysis revealed that elevated SIRI was significantly associated with female sex, larger tumor diameter, and higher Fuhrman grade (Table 1).

To evaluate prognostic value of preoperative SIRI in non-metastatic ccRCC, we performed Kaplan-Meier analysis to compare OS, CSS, and MFS between the high- and low-SIRI groups. The results demonstrated that elevated SIRI was significantly associated with worse OS, CSS and MFS (Fig. 2B-D). Furthermore, univariable and multivariable Cox proportional hazards models were employed, adjusting for potential confounders, including age, gender, hypertension, diabetes mellitus, coronary heart disease, body mass index (BMI), American society of Anesthesiologists (ASA) score, surgical approach, tumor laterality, multiple tumors, tumor size, pathologic T stage, Fuhrman grade, tumor hemorrhage and tumor necrosis. Multivariable analysis confirmed that SIRI served as an independent predictor of OS, CSS and MFS in patients with non-metastatic ccRCC (Table 2).

**Table 2:** Univariate and multivariate Cox proportional analysis of survival outcomes in 231 non-metastatic CcRCC patients

Parameters	OS				CSS				MFS			
	Univariate analysis		Multivariate analysis		Univariate analysis		Multivariate analysis		Univariate analysis		Multivariate analysis	
	HR (95% CI)	*P* value	HR (95% CI)	*P* value	HR (95% CI)	*P* value	HR (95% CI)	*P* value	HR (95% CI)	*P* value	HR (95% CI)	*P* value
Age	1.08 (1.04–1.13)	***< 0.001***	1.10 (1.05–1.16)	***< 0.001***	1.06 (1.02–1.11)	***0.010***	1.09 (1.03-0.15.03.15)	***0.003***	1.03(0.991.06)	***0.091***	-	-
Gender	1.05 (0.44–2.51)	***0.912***	-	-	0.92 (0.34–2.49)	***0.866***	-	-	0.98(0.481.99)	***0.945***	-	-
Hypertension	1.67 (0.72–3.88)	***0.231***	-	-	2.52 (0.93–6.82)	***0.070***	-	-	1.52(0.76–3.04)	***0.24***	-	-
Diabetes mellitus	1.91 (0.65–5.65)	***0.242***	-	-	1.80 (0.52–6.28)	***0.355***	-	-	1.62(0.62–4.22)	***0.321***	-	-
Coronary heart disease	2.41 (0.71–8.19)	***0.159***	-	-	0.98 (1.13–7.40)	***0.981***	-	-	0.99(0.24–4.18)	***0.997***	-	-
BMI	0.90 (0.78–1.02)	***0.099***	-	-	0.94 (0.81–1.09)	***0.396***	-	-	0.95(0.85–1.05)	***0.303***	-	-
ASA score	2.04 (0.94–4.46)	***0.072***	-	-	0.88 (0.31–2.49)	***0.810***	-	-	0.73(0.34–1.56)	***0.415***	-	-
Surgical approach	2.82 (0.66–12.10)	***0.164***	-	-	2.10 (0.48–9.23)	***0.327***	-	-	4.75(1.13–19.87)	***0.033***	2.61(0.61–11.16)	***0.196***
Tumor laterality	1.55 (0.65–3.70)	***0.326***	-	-	1.41 (1.21–1.640	***< 0.001***	-	-	1.21(0.60–2.44)	***0.586***	-	-
Multiple tumors	1.85 (0.25–13.83)	***0.55***	-	-	1.62 (0.60–4.40)	***0.340***	-	-	1.35(0.18–9.90)	***0.768***	-	-
Tumor size	1.30 (1.14–1.49)	***< 0.001***	1.18 (0.97–1.43)	***0.101***	2.27 (0.30–17.32.30.32)	***0.428***	1.34 (1.08–1.66)	***0.008***	1.36(1.20–1.54)	***< 0.001***	1.23(1.08–1.41)	***0.003***
Pathologic T stage	6.18 (2.09–18.31)	***< 0.001***	5.63 (1.74–18.20)	***0.004***	8.42 (2.74–25.87)	***< 0.001***	6.71 (2.01–22.37)	***0.002***	8.67(3.55–21.14)	***< 0.001***	5.43(2.17–13.59)	***< 0.001***
Fuhrman grade	6.61 (2.69–16.25)	***0.001***	2.10 (0.75–5.83)	***0.157***	4.55 (1.48–13.97)	***0.008***	1.26 (0.37–4.36)	***0.714***	3.13(1.20–8.17)	***0.019***	1.64 (0.61–4.40)	***0.328***
Tumor hemorrhage	0.78 (0.18–3.33)	***0.735***	-	-	1.02 (0.23–4.47)	***0.977***	-	-	1.11(0.39–3.16)	***0.85***	-	-
Tumor necrosis	1.11 (0.150–8.29)	***0.917***	-	-	1.41 (0.19–10.65)	***0.740***	-	-	1.58(0.38–6.61)	***0.533***	-	-
SIRI	5.32 (2.15–13.20)	***< 0.001***	4.61 (1.66–12.85)	***0.003***	4.98 (1.84–13.49)	***0.002***	3.82 (1.30–11.26.30.26)	***0.015***	3.90(1.94–7.86)	***< 0.001***	2.68 (1.31–5.49)	***0.007***

*BMI *Body mass index, *ASA *American society of Anesthesiologists, *SIRI *Systemic inflammation response index, *OS *Overall survival, *CSS *Cancer-specific survival, *MFS *Metastasis free survival, *HR *Hazard ratio, *95% CI *95%, confidence interval

### Prognostic nomogram construction and validation

Based on the established association between SIRI and survival outcomes in patients with non-metastatic ccRCC, we developed prognostic nomograms by integrating SIRI with clinicopathological variables. Variable selection was performed using LASSO regression, followed by multivariate Cox proportional hazards regression with backward elimination.

**Fig. 3:**
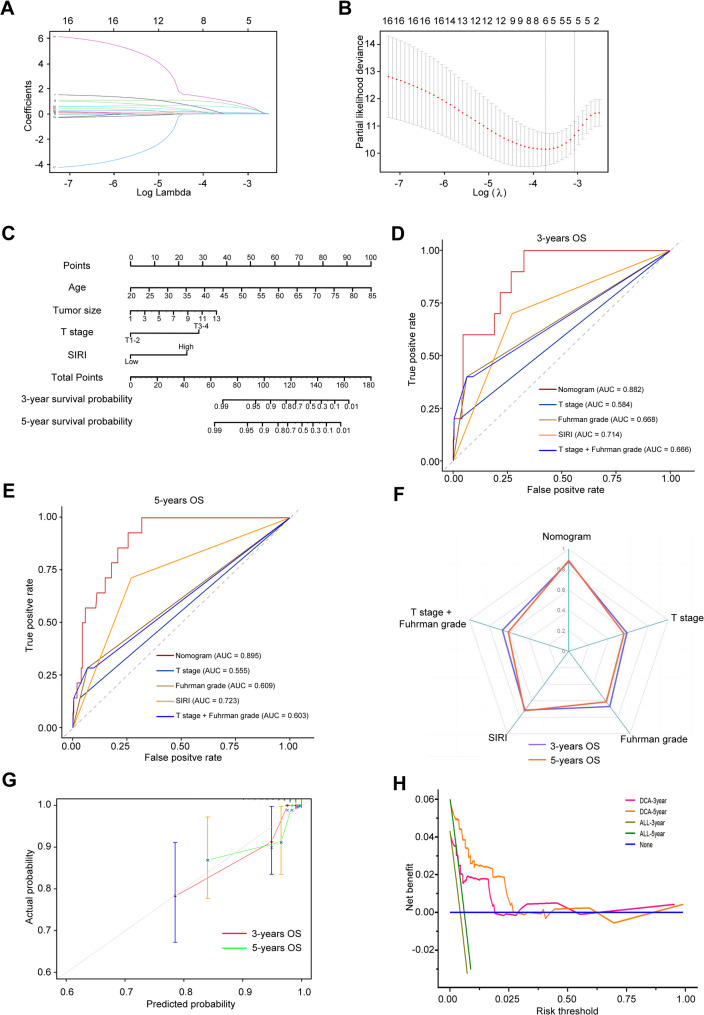
Development and validation of a prognostic nomogram for OS in patients with non-metastatic ccRCC integrating the SIRI and clinicopathological variables (**A)** LASSO regression analysis showing variable selection with log (λ) series; (**B**) Cross-validation curve for identifying the optimal λ value corresponding to the most regularized model within one standard error of the minimum binomial deviance. (**C**) Prognostic nomogram constructed using variables selected from LASSO and multivariable Cox regression to predict OS. (**D** and **E**) Time-dependent ROC curves evaluating the nomogram’s discriminative ability for 3-year (**D**) and 5-year (**E**) OS prediction. (**F**) C-index assessing discrimination of the nomogram. (**G**) Calibration curves of the nomogram comparing predicted and observed OS probabilities; dashed diagonal line represents ideal prediction. (**H**) DCA evaluating the clinical net benefit of the nomogram across different threshold probabilities. LASSO, Least absolute shrinkage and selection operator; C-index, Concordance index; DCA, Decision curve analysis

For OS, LASSO regression with λ.1se = 0.063 identified five significant predictors: age, tumor size, T stage, Fuhrman grade, and SIRI (Fig. 3A-B). Subsequent multivariate Cox analysis confirmed age, tumor size, T stage, and SIRI as independent prognostic factors (Table 3). The resulting OS nomogram (Fig. 3C) calculates cumulative risk scores based on these variables. The model demonstrated excellent discriminative ability, with 3- and 5-year AUCs values of 0.88 and 0.90, respectively (Fig. 3D-E; Table 4). and C-index of 0.88 and 0.89 (Fig. 3F; Table 4). Calibration curves revealed strong agreement between predicted and observed outcomes, and DCA confirmed favorable clinical utility (Fig. 3G-H).

**Table 3:** Multivariate analysis of parameters for the prediction of survival outcomes in 231 non-metastatic CcRCC patients

Parameters		OS			CSS			MFS	
	β	HR (95% CI)	*P* value	β	HR (95% CI)	*P* value	β	HR (95% CI)	*P* value
Age	0.10	1.11 (1.05–1.16)	***< 0.001***	-	-	***-***	-	-	***-***
Tumor size	0.19	1.22 (1.01–1.46)	***0.037***	1.49	1.28 (1.08–1.51)	***0.004***	0.23	1.26 (1.11–1.43)	***< 0.001***
T stage (T4 + T3/T2 + T1)	1.85	6.37 (2.00–20.32.00.32)	***0.002***	0.25	4.43 (1.38–14.19)	***0.012***	1.80	6.02 (2.41–15.01)	***< 0.001***
SIRI	1.51	4.54 (1.66–12.43)	***0.003***	1.27	3.55 (1.30–9.68)	***0.013***	1.03	2.81 (1.38–5.74)	***0.004***

*SIRI *Systemic inflammation response index, *OS *Overall survival, *CSS *Cancer-specific survival, *MFS *Metastasis free survival, *HR *Hazard ratio, *95% CI *95%, confidence interval

**Table 4:** AUC and C-index of the nomogram and other variables predicting survival outcomes

Parameters	AUC						Time C-index					
	3 Year			5 Year			3 Year			5 Year		
	OS	CSS	MFS	OS	CSS	MFS	OS	CSS	MFS	OS	CSS	MFS
Nomogram	0.88	0.83	0.78	0.90	0.81	0.77	0.88	0.83	0.77	0.89	0.81	0.76
T stage	0.58	0.63	0.60	0.56	0.57	0.62	0.59	0.63	0.60	0.56	0.58	0.62
Grade	0.67	0.61	0.55	0.61	0.56	0.56	0.67	0.62	0.55	0.61	0.56	0.56
SIRI	0.71	0.72	0.71	0.72	0.73	0.68	0.71	0.72	0.70	0.72	0.73	0.67
T stage + Grade	0.67	0.61	0.61	0.60	0.55	0.62	0.67	0.61	0.60	0.61	0.56	0.62

*SIRI *Systemic inflammation response index, *OS *Overall survival, *CSS *Cancer-specific survival, *MFS *Metastasis free survival, *AUC *Area under curve, *C-index *Concordance index

**Fig. 4:**
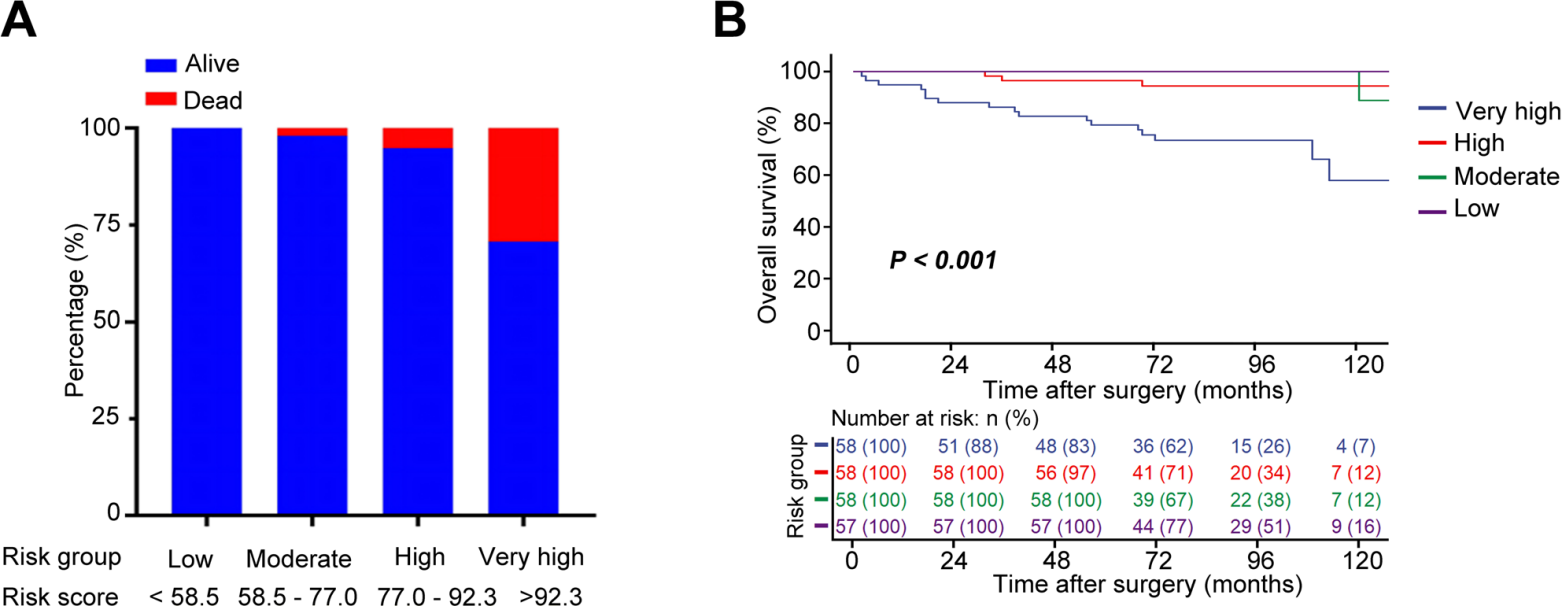
Risk stratification based on the OS nomogram in non-metastatic ccRCC patients (**A**) Number of all-cause deaths across four risk groups (Low, Moderate, High, and Very High) stratified by quartiles of the nomogram-predicted risk score. **B** Kaplan–Meier curves comparing OS probabilities among the four risk groups

Using interquartile-derived cutoffs, patients were stratified into four OS risk groups: low-risk (< 58.5), moderate-risk (58.5–77), high-risk (77–92.3), and very high-risk (> 92.3) (Supplementary Tables 1–2, Fig. 4A). Kaplan-Meier analysis revealed significantly poor survival in higher-risk groups (Fig. 4B).

**Fig. 5:**
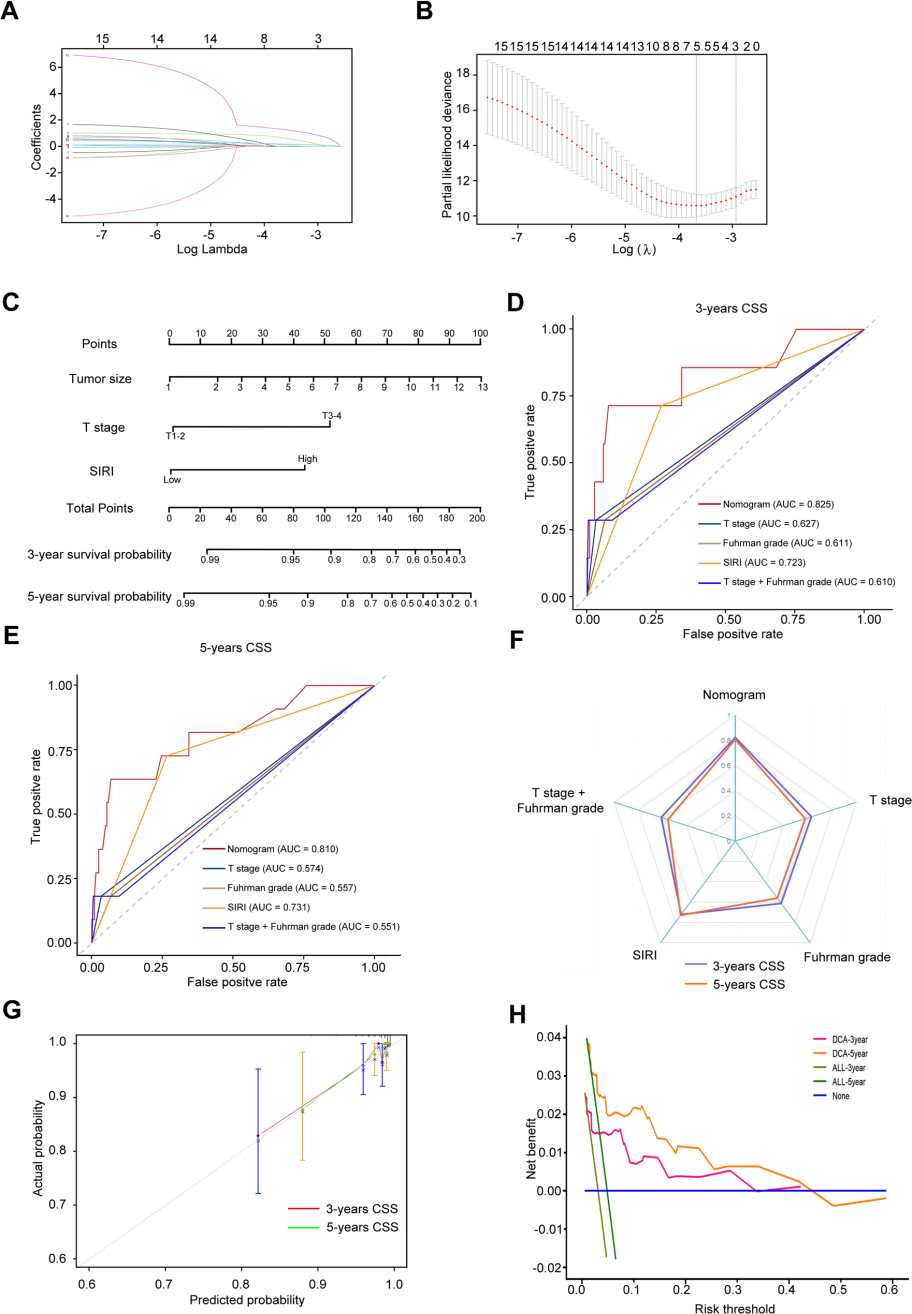
Development and validation of a prognostic nomogram for CSS in patients with non-metastatic ccRCC integrating the SIRI and clinicopathological variables (**A)** LASSO regression analysis showing variable selection with log (λ) series; (**B**) Cross-validation curve for identifying the optimal λ value corresponding to the most regularized model within one standard error of the minimum binomial deviance. **C** Prognostic nomogram constructed using variables selected from LASSO and multivariable Cox regression to predict CSS. **D** and **E** Time-dependent ROC curves evaluating the nomogram’s discriminative ability for 3-year (**D**) and 5-year (**E**) CSS prediction. **F** C-index assessing discrimination of the nomogram. **G** Calibration curves of the nomogram comparing predicted and observed CSS probabilities; dashed diagonal line represents ideal prediction. **H** DCA evaluating the clinical net benefit of the nomogram across different threshold probabilities

**Fig. 6:**
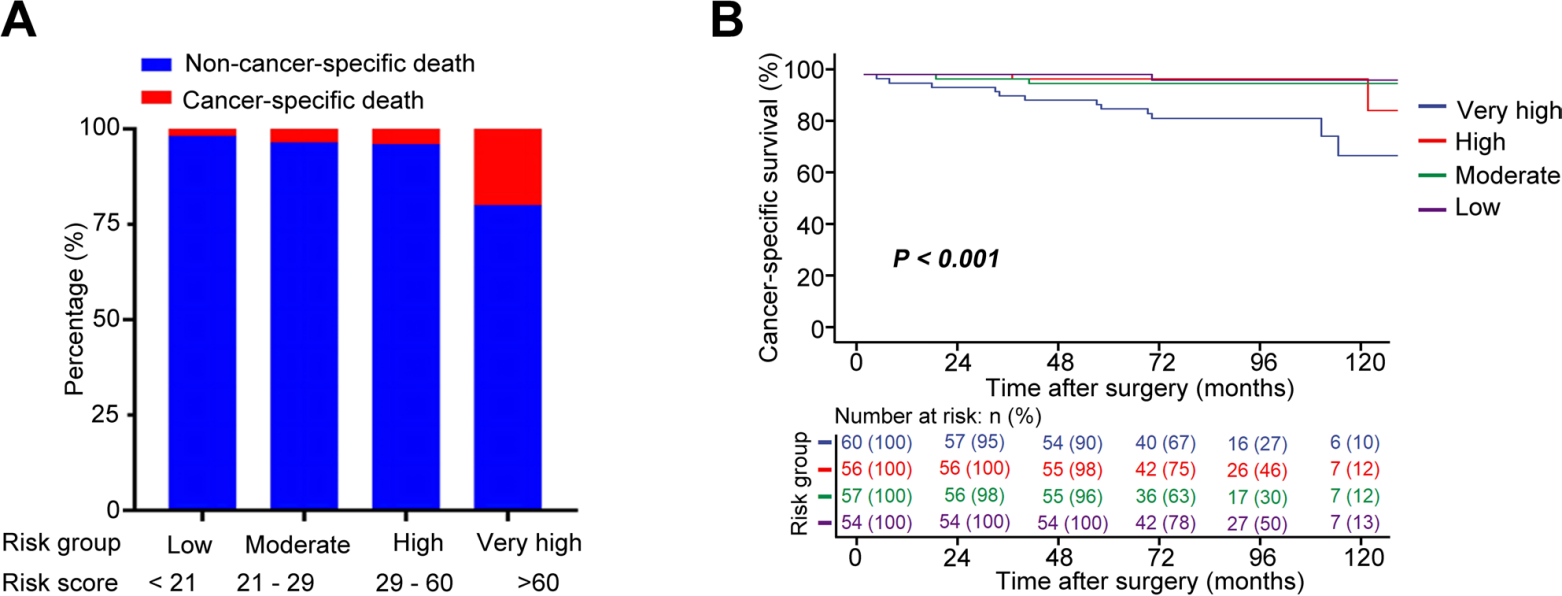
Risk stratification based on the CSS nomogram in non-metastatic ccRCC patients (**A**) Number of cancer-specific deaths across four risk groups (Low, Moderate, High, and Very High) stratified by quartiles of the nomogram-predicted risk score. **B** Kaplan–Meier curves comparing CSS probabilities among the four risk groups

For CSS, LASSO regression (λ.1se = 0.040) and multivariate Cox analysis identified tumor size, T stage, and SIRI as independent predictors (Fig. 5A-B; Table 3). The CSS prognostic nomogram (Fig. 5C) achieved 3- and 5-year AUCs of 0.83 and 0.81 (Fig. 5D-E; Table 4) with C-index of 0.83 and 0.81, respectively (Fig. 5F; Table 4). Calibration and DCA curves demonstrated robust model performance (Fig. 5G-H). Risk stratification based on interquartile ranges (low: <21; moderate: 21–29; high: 29–60; very high: >60) revealed progressively worse CSS in higher-risk groups (Supplementary Tables 1–2, Fig. 6A-B).

**Fig. 7:**
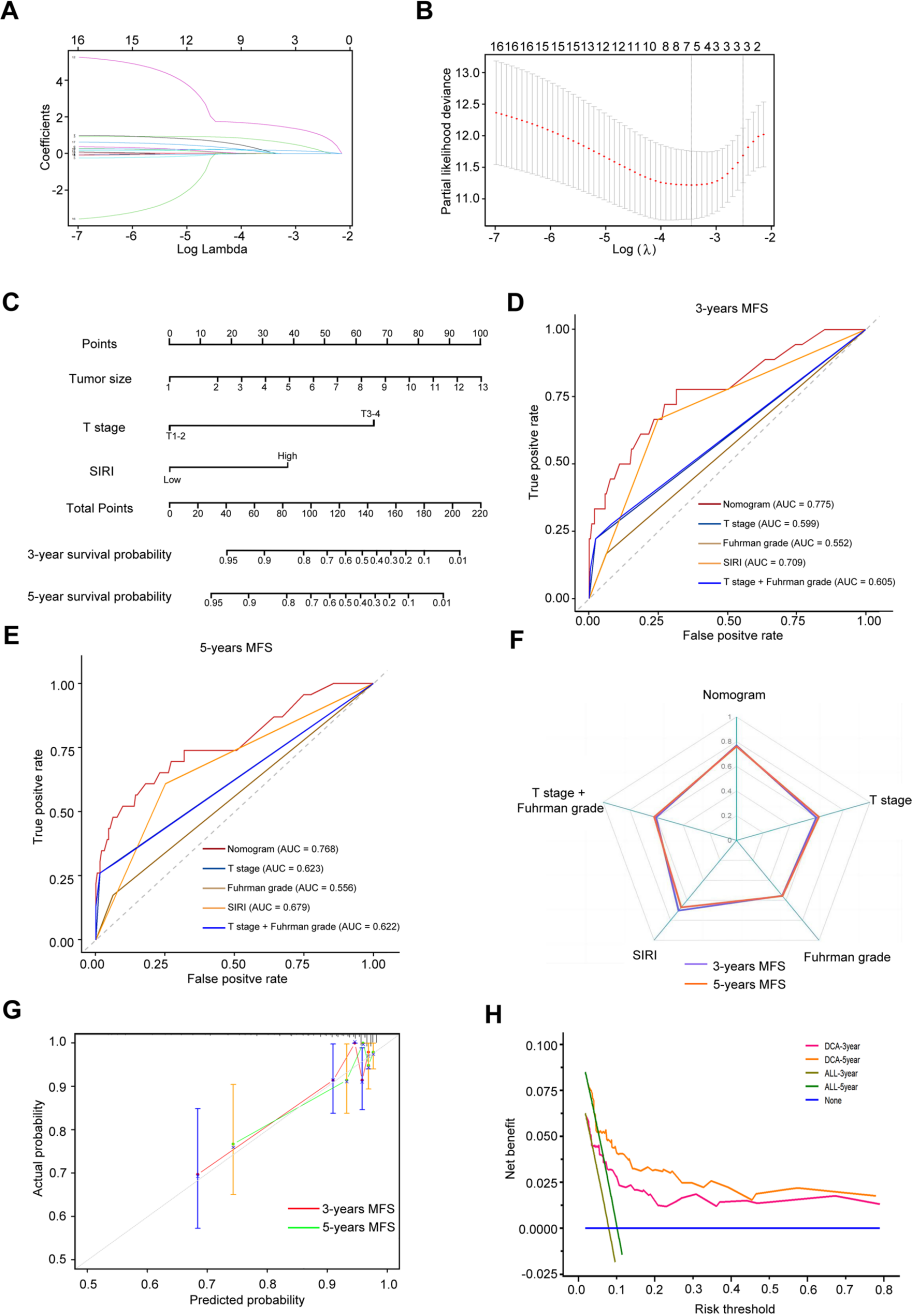
Development and validation of a prognostic nomogram for MFS in patients with non-metastatic ccRCC integrating the SIRI and clinicopathological variables (**A)** LASSO regression analysis showing variable selection with log (λ) series; (**B**) Cross-validation curve for identifying the optimal λ value corresponding to the most regularized model within one standard error of the minimum binomial deviance. **C** Prognostic nomogram constructed using variables selected from LASSO and multivariable Cox regression to predict MFS. **D** and **E** Time-dependent ROC curves evaluating the nomogram’s discriminative ability for 3-year (**D**) and 5-year (**E**) MFS prediction. **F** C-index assessing discrimination of the nomogram. **G** Calibration curves of the nomogram comparing predicted and observed MFS probabilities; dashed diagonal line represents ideal prediction. **H** DCA evaluating the clinical net benefit of the nomogram across different threshold probabilities

**Fig. 8:**
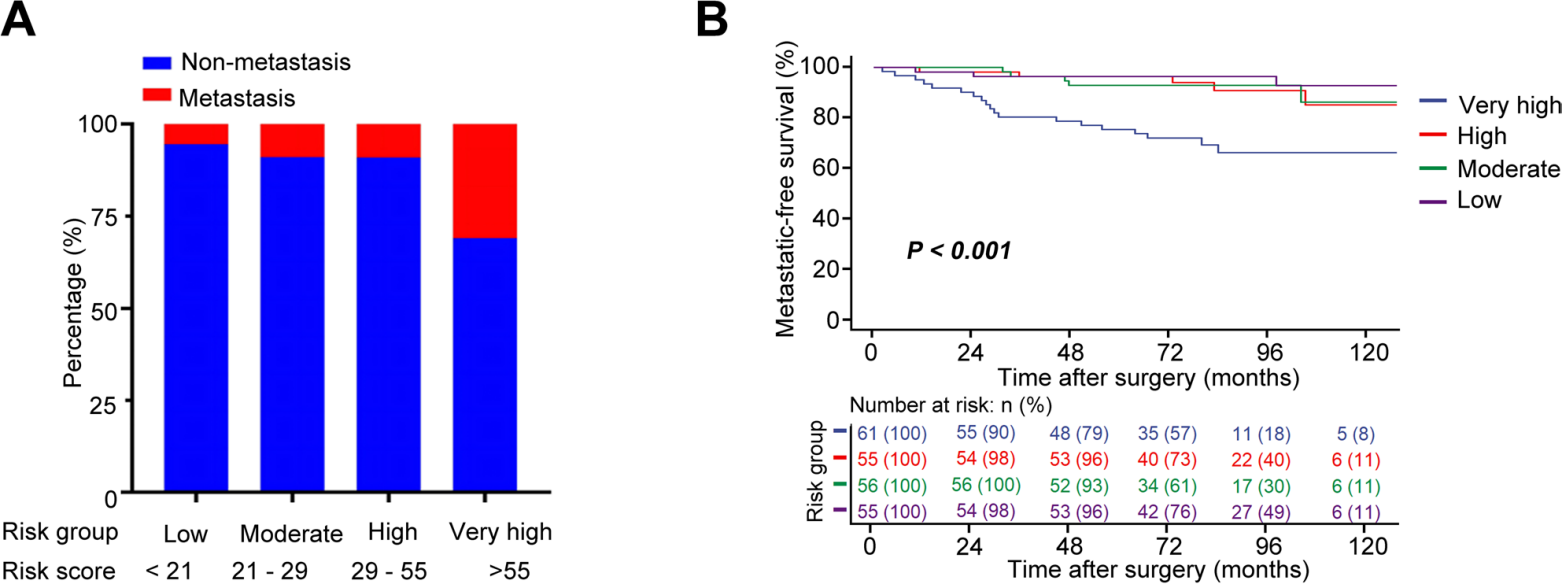
Risk stratification based on the MFS nomogram in non-metastatic ccRCC patients (**A**) Number of metastasis across four risk groups (Low, Moderate, High, and Very High) stratified by quartiles of the nomogram-predicted risk score. **B** Kaplan–Meier curves comparing MFS probabilities among the four risk groups

For MFS, tumor size, T stage, and SIRI emerged as significant predictors (λ.1se = 0.035; Fig. 7A-B; Table 3). The MFS prognostic nomogram (Fig. 7C) yielded 3- and 5-year AUCs of 0.78 and 0.77 (Fig. 7D-E; Table 4), and C-indices of 0.77 and 0.76 (Fig. 7F; Table 4), supported by strong calibration and clinical utility (Fig. 7G-H). Stratification into low- (< 21), moderate- (21–29), high- (29–55), and very high (> 55) groups showed significantly reduced MFS in higher-risk categories (Supplementary Tables 1–2, Fig. 8A-B).

### Compared with established models

**Fig. 9:**
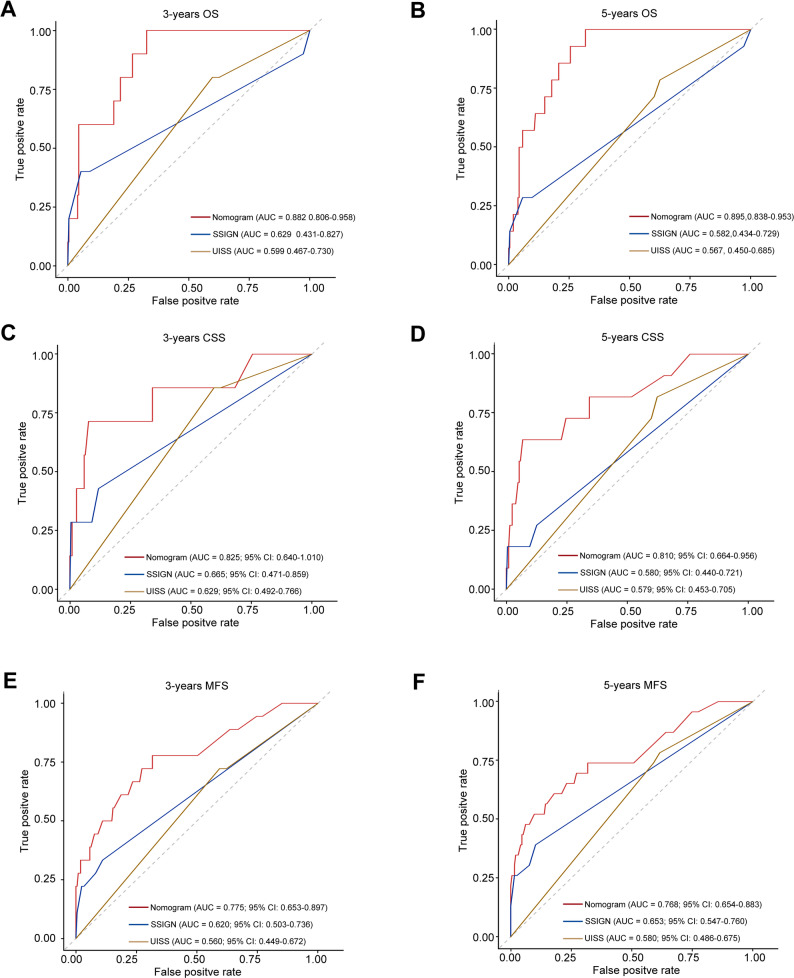
Comparison of prognostic performance between the proposed nomogram, SSIGN score, and UISS using time-dependent ROC analysis in non-metastatic ccRCC patients (**A** and **B**) Discriminative ability for predicting 3‑year (**A**) and 5‑year (**B**) OS. **C** and **D** Discriminative ability for 3‑year (**C**) and 5‑year (**D**) CSS. **E** and **F** Discriminative ability for 3‑year (**E**) and 5‑year (**F**) MFS. SSIGN, Stage, Size, Grade, and Necrosis score; UISS, University of California Los Angeles Integrated Staging System

We further evaluated the prognostic accuracy of our nomogram by comparing it with two widely used models in RCC: the SSIGN score and UISS. Comparative analyses demonstrated that our nomogram exhibited significantly superior discriminative ability for predicting 3- and 5-year survival outcomes compared to both the SSIGN and UISS models (Fig. 9A-F). Collectively, these results affirm the enhanced risk-stratification capability of our nomogram in patients with non-metastatic ccRCC.

## Discussion

Cancer patients are known to exhibit altered systemic inflammatory responses [12] which play critical roles throughout tumor progression [5]. RCC is recognized as one of the most immunologically active malignancies [13]. Based on this concept, the SIRI, defined by peripheral neutrophil, lymphocyte, and monocyte counts, was initially established by Qi et al. as a reliable prognostic indicator [9]. In the present study, SIRI was identified as an independent predictor of OS, CSS, and metastatic progression in patients with non-metastatic ccRCC. We further integrated SIRI with clinicopathological parameters to develop and validate prognostic nomograms for OS, CSS, and metastasis. Comparative analysis against established models demonstrated that our prognostic nomogram exhibited statistically superior predictive accuracy.

The prognostic significance of SIRI in tumors may be elucidated by several underlying mechanisms. Neutrophils, as core components of SIRI, influence prognosis across multiple malignancies and participate in all phases of tumor progression, including in ccRCC [14]. Tumor associated neutrophils exhibit functional duality: the N1 phenotypes exerts anti-tumor activity, whereas N2 phenotypes promote tumorigenesis [15, 16]. The phenotypic shift from N1 to N2 is regulated by TGF-β, which is derived from either tumor cells or the tumor microenvironment [14, 16]. Lymphocyte also play a central role in tumor development and represent a major focus in contemporary oncology research. CD8^+^ T lymphocytes act as primary cytotoxic effectors capable of directly eliminating tumor cells, while CD4^+^ T helper lymphocytes facilitate the activation of cytotoxic T lymphocytes (CTLs) [17]. In contrast, FoxP3^+^ regulatory T lymphocytes suppress CTL-mediated anti-tumor responses. Furthermore, monocyte dynamics are reprogrammed in cancer patients through alterations in systemic cytokines, such as colony-stimulating factor 1 (CSF1), CSF2 and CSF3 [18]. Within this inflammatory microenvironment, direct contact between monocytes and tumor cells or tumor-drived factors further promotes functional adaptations in circulating monocyte subsets, thereby contributing to tumor progression.

In 2019, Chen et al. [10] evaluated the prognostic utility of the SIRI in a cohort of 414 patients with localized or locally advanced ccRCC. Their results demonstrated that patients with an elevated SIRI had significantly worse OS and CSS compared to those with lower SIRI. Multivariate analysis confirmed SIRI as an independent predictor for both OS (HR = 4.85; 95% CI 2.36–9.98; *P* < 0.001) and CSS (HR = 5.91; 95% CI 2.68–13.04; *P* < 0.001). Even after propensity score matching, SIRI remained a robust predictor for both endpoints and exhibited superior discriminative ability compared to the PLR, NLR, MLR, and the Memorial Sloan Kettering Cancer Center prognostic score. However, Chen et al. focused solely on the prognostic value of SIRI in ccRCC and did not combine it with traditional clinicopathological prognostic factors. Our study bridges this gap by developing and rigorously validating a nomogram that integrates SIRI with key clinicopathological variables. In 2021, Mao et al. [11] analyzed 443 patients with RCC and found that SIRI had superior predictive performance for OS and CSS compared to the LMR and hemoglobin level. They also developed a prognostic nomogram based on SIRI. It should be noted, however, that their calculation of SIRI incorporated hemoglobin and LMR rather than the conventional neutrophil, lymphocyte, and monocyte counts. Moreover, their study cohort encompassed all RCC subtypes, while our study specifically targets patients with non-metastatic ccRCC. A meta-analysis of 14 studies involving 3,744 RCC patients demonstrated that the systemic immune index, calculated from peripheral lymphocyte, neutrophil, and platelet counts, is associated with poor prognosis [4]. However, this analysis did not evaluate the ability of inflammatory biomarkers to predict metastasis. Our study addresses this unmet need by establishing SIRI as a significant predictor of metastatic progression in patients with non-metastatic ccRCC, thereby providing novel insights to the field.

The integration of systemic inflammation markers into prognostic models has been explored in previous studies, highlighting their potential to improve predictive accuracy. For instance, Allenet et al. demonstrated that incorporating the NLR into the UISS model enhanced its predictive efficacy for recurrence in localized renal cell carcinoma [19]. Similarly, Hutterer et al. reported that adding the LMR to the Leibovich prognosis score enhanced its ability to predict recurrence risk after nephrectomy [20]. Inspired by this foundation, we developed a novel prognostic model that combines the SIRI with key clinicopathological parameters. This integrated model demonstrated superior predictive performance for OS, CSS, and MFS in patients with non-metastatic ccRCC compared to the individual parameters alone. Furthermore, when evaluated against established RCC prognostic models such as SSIGN and UISS, our model exhibited significantly enhanced predictive performance.

In this study, patients were categorized into four distinct risk groups based on the risk score derived scores. Although the same set of predictors was used in the models for OS, CSS, and MFS models, the weightings assigned to each factor varied depending on the clinical endpoint. Kaplan-Meier analysis revealed a significantly higher incidence of all-cause mortality, cancer specific death, and metastatic progression among patients with higher nomogram scores. This stratification approach offers a practical framework to support clinical risk assessment and decision-making.

Our study presents several notable strengths. First, the SIRI is calculated from routine blood parameters, offering a cost-effective approach for risk stratification that does not require additional expensive tests. Second, to our knowledge, this is the first study to develop and validate comprehensive nomograms for predicting OS, CSS, and MFS specifically in patients with non-metastatic ccRCC, with demonstrated superior predictive performance over existing models. Finally, all predictors included in the nomograms are clinically practical, being non-invasive, easily obtainable, highly reproducible, and cost-efficient.

Our study has several limitations that warrant consideration. First, the retrospective single-center design and moderate sample size may introduce potential selection bias and restrict generalizability of our findings. It is also important to note that T-stage and tumor size are known to be correlated, as tumor size is a key component in determining T-stage. However, T-stage was ultimately selected for the final model due to its comprehensive incorporation of local invasion and its established role in standardized clinical staging and practice. Additionally, the inflammatory markers such as SIRI were retrospectively obtained from preoperative laboratory data, which may be subject to unstandardized variations despite adherence to institutional protocols. Furthermore, SIRI and other hematologic biomarkers can be influenced by non-oncologic conditions, including undetected infections, chronic inflammatory diseases, or the use of medications such as corticosteroids and other immunosuppressive agents. Another consideration is that study period spanned from 2011 to 2017, and ongoing therapeutic advances may affect contemporary applicability for our results. Therefore, the prognostic performance and applicability of our model in patients receiving current standard treatments require further validation. To address these limitations, we recommend external validation through multi-institutional and prospective studies to verify generalizability across diverse populations. The incorporation of novel imaging biomarkers and molecular profiling may also improve prognostic accuracy. Moreover, practical implementation challenges, such as the need for specialized software, clinician training, and integration with electronic health records, must be addressed to facilitate the translation of these nomograms into routine clinical use. Therefore, we emphasize that prospective validation and multimodal data integration are essential next steps toward developing personalized clinical decision-support tools based on our findings.

## Conclusion

In summary, elevated pretreatment SIRI was significantly associated with worse OS, CSS, and MFS in patients with non-metastatic ccRCC. The prognostic nomogram integrating SIRI with key clinicopathological parameters shows potential for improving postoperative risk stratification, although prospective validation is necessary before it can be adopted into clinical practice.

## Supplementary Information

Supplementary Material 1.

Supplementary Material 2.

## Data Availability

The original data of the present study were available from the corresponding author on reasonable requests.

## Abbreviations

AJCC: American Joint Committee on Cancer
AUC: Area under curve
SIRI: Systemic inflammation response index
OS: Overall survival
CSS: Cancer specific survival
MFS: Metastasis free survival
ccRCC: Clear cell renal cell carcinoma
RCC: Renal cell carcinoma
ROC: Receiver operating characteristic curve
C- index: Concordance index
DCA: Decision curve analysis
SSIGN: Stage, Size, Grade, and Necrosis score
UISS: University of California Los Angeles Integrated Staging System
CT: Computed tomography
MRI: Magnetic resonance imaging
PET-CT: Positron emission tomography–CT
SD: Standard deviation
IQR: Interquartile range
C-index: Concordance index
BMI: Body mass index
ASA: American society of Anesthesiologists score
CTLs: Cytotoxic T lymphocytes
CSF: Colony stimulating factor
PLR: Platelet to lymphocyte ratio
NLR: Neutrophil to lymphocyte ratio
MLR: Monocyte to lymphocyte ratio
LMR: Lymphocyte to monocyte ratio
LASSO: Least absolute shrinkage and selection operator
