# Supplementary material for: Nomograms based on SIRI for predicting postoperative survival outcomes in patients with non-metastatic clear cell renal cell carcinoma
Source: BMC Surg. 2025 Dec 24;25:593. doi: 10.1186/s12893-025-03349-y (PMC12729437; doi:10.1186/s12893-025-03349-y)
Supplement: Supplementary file 1 — Supplementary Material 1. [file 12893_2025_3349_MOESM1_ESM.docx]

| Supplementary Table 1.Scores of parameters for the prediction of survival outcomes in ccRCC patients | | | | | | |
| --- | --- | --- | --- | --- | --- | --- |
| Parameters | Category | Point | | | | |
|  |  | OS Score |  | CSS Score |  | MFS Score |
| Age, years |  |  |  |  |  |  |
|  | 20 | 0 |  |  |  |  |
|  | 25 | 8 |  |  |  |  |
|  | 30 | 15 |  |  |  |  |
|  | 35 | 23 |  |  |  |  |
|  | 40 | 31 |  |  |  |  |
|  | 45 | 38 |  |  |  |  |
|  | 50 | 46 |  |  |  |  |
|  | 55 | 54 |  |  |  |  |
|  | 60 | 62 |  |  |  |  |
|  | 65 | 69 |  |  |  |  |
|  | 70 | 77 |  |  |  |  |
|  | 75 | 85 |  |  |  |  |
|  | 80 | 92 |  |  |  |  |
|  | 85 | 100 |  |  |  |  |
| Tumor size,cm | 1 | 0 |  | 0 |  | 0 |
|  | 2 | 3 |  | 8 |  | 8 |
|  | 3 | 6 |  | 17 |  | 17 |
|  | 4 | 9 |  | 25 |  | 25 |
|  | 5 | 12 |  | 33 |  | 33 |
|  | 6 | 15 |  | 42 |  | 42 |
|  | 7 | 18 |  | 50 |  | 50 |
|  | 8 | 21 |  | 58 |  | 58 |
|  | 9 | 24 |  | 67 |  | 67 |
|  | 10 | 27 |  | 75 |  | 75 |
|  | 11 | 30 |  | 83 |  | 83 |
|  | 12 | 33 |  | 92 |  | 92 |
|  | 13 | 36 |  | 100 |  | 100 |
| Pathologic T stage | T1+T2 | 0 |  | 0 |  | 0 |
|  | T3+T4 | 29 |  | 51 |  | 66 |
| SIRI | <1.405 | 0 |  | 0 |  | 0 |
|  | >1.405 | 23 |  | 43 |  | 38 |
