# Supplementary material for: Nomograms based on SIRI for predicting postoperative survival outcomes in patients with non-metastatic clear cell renal cell carcinoma
Source: BMC Surg. 2025 Dec 24;25:593. doi: 10.1186/s12893-025-03349-y (PMC12729437; doi:10.1186/s12893-025-03349-y)
Supplement: Supplementary file 2 — Supplementary Material 2. [file 12893_2025_3349_MOESM2_ESM.docx]

| \| Supplementary Table2. Survival results for different risk subgroups \| \| \| \| \| \| \| \| \| \| \| --- \| --- \| --- \| --- \| --- \| --- \| --- \| --- \| --- \| --- \| \|  \| Survival \| \| P \| Cancer-specific death \| \| P \| Metastasis \| \| P \| \| Risk group \| Alive (n=210) \| Dead (n=22) \| <0.001 \| No (n=210) \| Yes (n=17) \| <0.001 \| No (n=210) \| Yes (n=17) \| <0.001 \| \| Low \| 57(%) \| 0 \|  \| 53(%) \| 1 \|  \| 52(%) \| 3(%) \|  \| \| Moderate \| 57(%) \| 1(%) \|  \| 55(%) \| 2(%) \|  \| 51(%) \| 5(%) \|  \| \| High \| 55(%) \| 3(%) \|  \| 54(%) \| 2(%) \|  \| 50(%) \| 5(%) \|  \| \| Very high \| 41(%) \| 17(%) \|  \| 48(%) \| 12(%) \|  \| 52(%) \| 19(%) \|  \| \| OS risk group,Low:<58.5, Moderate:58.5-77, High:77-92.3, Very high:>92.3; \| \| \| \| \| \| \| \| \| \| \| CSS risk group,Low:<21, Moderate:21-29, High:29-60, Very high:>60; \| \| \| \| \| \| \| \| \| \| \| MFS risk group,Low:<21, Moderate:21-29, High:29-55, Very high:>55. \| \| \| \| \| \| \| \| \| \| |
| --- | --- | --- | --- | --- | --- | --- | --- | --- | --- | --- | --- | --- | --- | --- | --- | --- | --- | --- | --- | --- | --- | --- | --- | --- | --- | --- | --- | --- | --- | --- | --- | --- | --- | --- | --- | --- | --- | --- | --- | --- | --- | --- | --- | --- | --- | --- | --- | --- | --- | --- | --- | --- | --- | --- | --- | --- | --- | --- | --- | --- | --- | --- | --- | --- | --- | --- | --- | --- | --- | --- | --- | --- | --- | --- | --- | --- | --- | --- | --- | --- | --- | --- | --- | --- | --- | --- | --- | --- | --- | --- | --- | --- | --- | --- | --- | --- | --- | --- | --- | --- |
